# Editorial: Salicylic Acid Signaling Networks

**DOI:** 10.3389/fpls.2016.00238

**Published:** 2016-02-24

**Authors:** Hua Lu, Jean T. Greenberg, Loreto Holuigue

**Affiliations:** ^1^Department of Biological Sciences, University of Maryland Baltimore CountyBaltimore, MD, USA; ^2^Department of Molecular Genetics and Cell Biology, The University of ChicagoChicago, IL, USA; ^3^Departamento de Genética Molecular y Microbiología, Facultad de Ciencias Biológicas, Pontificia Universidad Católica de ChileSantiago, Chile

**Keywords:** crosstalk, systemic acquired resistance, flowering, circadian clock, SA receptor, reactive oxygen species, effector, NPR1

The small phenolic compound salicylic acid (SA) is critical for plant defense against a broad spectrum of pathogens and responses to different abiotic stress conditions. Particularly in response to pathogens, SA is involved in multiple processes, including basal and resistance gene-mediated defense as well as systemic acquired resistance (SAR). This Research Topic includes a collection of 18 articles for reviews, perspectives, and original research, to highlight recent exciting progress toward our understanding of molecular mechanisms underlying SA-mediated defense and SA-crosstalk to other pathways.

Seyfferth and Tsuda summarize the regulation of SA levels, perception, and transcriptional reprogramming (Seyfferth and Tsuda). Besides SA biosynthetic enzymes, the SA levels can be affected by multiple mechanisms mediated by some non-enzyme proteins (Lu, 2009; Dempsey et al., 2011). One of such mechanisms depends on calcium signaling. The calmodulin-binding transcription factor CBP60g and its close homolog SARD1 control expression of the SA biosynthetic gene *ICS1*, highlighting a role of calcium signaling in initiating SA synthesis (Seyfferth and Tsuda).

For SA-mediated transcriptional reprogramming, NPR1 has been demonstrated as a master co-activator that interacts with bZIP transcription factors in the TGA family (Seyfferth and Tsuda; Yan and Dong, 2014). SA controls NPR1 function by regulating its protein level in the nucleus, mainly through posttranslational modifications (Mou et al., 2003; Tada et al., 2008). Furniss and Spoel review the roles of ubiquitin-mediated protein degradation and sumoylation in modulating NPR1 function (Furniss and Spoel; Saleh et al., 2015). Recently two NPR1 homologs, NPR3 and NPR4, were shown to be SA receptors that have different SA-binding affinities and target NPR1 for ubiquitin-mediated protein degradation under high and low SA conditions, respectively (Fu et al., 2012). The primary working condition for NPR1 requires intermediate SA levels. Thus, creating SA gradient in the defense zone is critical for SA signaling. Interestingly, whether or not NPR1 itself is an SA receptor has been controversial (Fu et al., 2012; Wu et al., 2012). A perspective article compares SA-binding properties of NPR1, NPR3, and NPR4 under different laboratory conditions (Kuai et al.). Such information should help to clarify the controversy and highlight the possibility of NPR1 as another SA receptor. However, questions still remain about how multiple SA receptors coordinate with each other to transduce SA perception into signaling and ultimately transcriptional reprogramming.

A localized foliar infection of plants can lead to SAR, a long lasting resistance against a broad spectrum of pathogens at the systemic level. Gao and coworkers summarize the importance of SA in establishing SAR in plants (Gao et al.). Some mutants impaired in SA accumulation and/or signaling are compromised in SAR (Gao et al.). At least one SA derivative, methyl SA has been implicated in SAR (Park et al., 2007). Some SAR-inducing molecules require SA for the establishment or manifestation of SAR. For example, the SAR molecule azelaic acid acts by priming elevated SA production upon secondary infection (Jung et al., 2009). In addition, treating plants with the SAR-related molecule diterpenoid dehydrobietinal leads to SA accumulation in the absence of pathogen infection (Chaturvedi et al., 2012).

Given the critical roles of SA in plant defense and our lack of a complete understanding of SA signaling, it is important to uncover additional genes involved in SA-mediated defense. Two mutant screens are reported in this Research Topic for this purpose (Ding et al.; Manohar et al.). To look for SA binding proteins, Monahar and coworkers used a photo-reactive SA analog 4-AzidoSA (4AzSA) in a protein microarray (Manohar et al.). To look for genes affecting SA levels, Ding and coworkers used a biosensor-based method (Ding et al.). Different from some previous screens, these two screens were conducted at a large scale with high throughput and are anticipated to discover new and uncharacterized SA-related genes besides the ones that are already known.

While clearly representing a hub in plant defense signaling networks, SA is also known to exhibit crosstalk with other signaling pathways, such as those mediated by some phytohormones and reactive oxygen species (ROS). The antagonistic and synergistic relationship between SA and the phytohormone jasmonic acid (JA) is the focus of many discussions. Caarls and colleagues review the molecular mechanisms underlying transcriptional control of JA-induced genes by SA (Caarls et al.). The crosstalk between SA and JA is also dependent on the redox status of cells controlled by the TRX/GRX oxidoreductase enzymes as discussed by Herrera-Vasquez et al. Some SA transcriptional regulators, i.e., NPR1 and TGAs, are redox sensors and can be directly or indirectly affected by some TRX/GRX oxidoreductase enzymes, highlighting the interplay between SA, JA, and redox signaling (Caarls et al.; Herrera-Vasquez et al.). The research article by Westlake and co-workers reports a redox-sensing function of two SA binding proteins, TOP1 and TOP2, further underscoring the importance of ROS in SA signaling (Westlake et al.).

The crosstalk between SA and lipids is discussed in a collection of four papers in this Research Topic. Sanchez-Rangel and coworkers review the role of sphingolipids affecting SA accumulation (Sanchez-Rangel et al.). On the other hand, the research paper by Shi and coworkers show that SA could reciprocally influence the sphingolipid profile, using *in silico* Flux Balance Analysis and experimental validation (Shi et al.). The roles of two phospholipids, phosphatidic acid (PA) and phosphatidylinositol 4-phosphate, in affecting SA-mediated defense are reviewed by Zhang and Xiao. Janda and co-workers further show that one possible mechanism of PA function in SA defense is through affecting NPR1 localization (Janda et al.).

Emerging evidence shows that there is crosstalk between SA and the circadian clock, the internal time measuring machinery of plants to ensure growth, development, and proper responses to stresses. The circadian clock controls diurnal biosynthesis of SA and SA also feedback regulates clock activity (Goodspeed et al., 2012; Zheng et al., 2015; Zhou et al., 2015). The research article by Wang and co-workers reports a possible direct regulation of the defense gene *PHT4;1* by the core clock gene *CCA1* (Wang et al.), providing a potential molecular link for clock-defense crosstalk.

Crosstalk of SA to many signaling pathways suggests that SA could affect multiple cellular processes besides its central role in controlling immunity. Two articles in this Research Topic discuss the role of SA in affecting plant development with a focus on leaf senescence and flowering time control (Banday and Nandi; Carella et al.). Carella and coworkers also report that SA and some gene components in the SA pathway contribute to age-related resistance, a form of developmentally regulated pathogen resistance of plants (Carella et al.).

Because of the key role of SA in host defense activation, it is not surprising that the SA hub is hijacked by many pathogens in order to promote pathogen virulence and induce host susceptibility (Caarls et al.; Tanaka et al.). Bacterial and fungal pathogens are known to deliver effector proteins to the host cell and affect SA metabolism, SA signaling, and SA crosstalk with the JA pathway. It is not known yet though if pathogen effectors could bind directly to SA biosynthetic enzyme(s) and/or signaling proteins to modulate their activities and subsequently lead to altered SA levels and/or signaling. Besides effector proteins, pathogens can also produce chemicals to mimic host compounds in order to interfere with host signaling pathways. For instance, coronatine (COR) produced by *Pseudomonas syringae* is structurally similar to JA-Ile (the active form of JA). COR can activate host JA pathway and subsequently suppress SA accumulation and signaling (Zheng et al., 2012). Interestingly, while pathogens can use effectors and/or chemicals to target the SA hub for their own benefit, the host can also recognize some pathogen effectors and/or chemicals and subsequently activate strong defense responses to fight against the invaders. For instance, plant recognition of a cognate avirulence effector by a resistance protein activates much stronger and faster SA and ROS accumulation and cell death, leading to enhanced disease resistance (Hamdoun et al., 2013). In addition, plants treated with quorum sensing molecules, such as N-acyl homoserine lactones, are primed for stronger and faster defense activation upon further defense challenge (Baumgardt et al., 2014; Schenk and Schikora). Such defense priming is dependent on SA, JA, and JA related metabolites.

The articles collected in this Research Topic represent our current understanding of multifaceted function of SA and the complexity of SA signaling networks. They will serve as a catalyst for further discussions and discoveries. Many exciting advances are expected to come in the near future, such as identification of new players in the SA signaling networks, elucidation of molecular mechanisms underlying the crosstalk of SA with other pathways, and discovery of pathogen effectors that directly target SA pathway genes and proteins. The central role of SA in plant defense and its crosstalk to other physiological processes make it critically important to further understand SA signaling networks. Manipulation of genes on the SA signaling networks provides a promising way to enhance disease resistance in economically important plants.

## Author contributions

All authors listed, have made substantial, direct and intellectual contribution to the work, and approved it for publication.

### Conflict of interest statement

The authors declare that the research was conducted in the absence of any commercial or financial relationships that could be construed as a potential conflict of interest.
